# Occurrence and genetic characterization of *Enterocytozoon bieneusi* in pet dogs in Yunnan Province, China[Fn FN1]

**DOI:** 10.1051/parasite/2024025

**Published:** 2024-05-24

**Authors:** Jinhua Jian, Jinrong Zi, Yaxue Wang, Yaming Yang, Yaxing Su, Lan Yao, Benfu Li, Xiaoxue Peng, Jianping Cao, Yujuan Shen, Aiqin Liu

**Affiliations:** 1 Hangzhou Medical College Hangzhou 310000 PR China; 2 National Key Laboratory of Intelligent Tracking and Forecasting for Infectious Diseases, National Institute of Parasitic Diseases, Chinese Center for Disease Control and Prevention (Chinese Center for Tropical Diseases Research), NHC Key Laboratory of Parasite and Vector Biology, WHO Collaborating Centre for Tropical Diseases, National Center for International Research on Tropical Diseases Shanghai 200025 PR China; 3 Department of Helminth, Yunnan Institute of Parasitic Diseases Puer 655099 PR China; 4 Department of Parasitology, Harbin Medical University Harbin 150081 PR China

**Keywords:** *Enterocytozoon bieneusi*, Genotyping, Dogs, Zoonotic transmission

## Abstract

*Enterocytozoon bieneusi* is the most common microsporidian species in humans and can affect over 200 animal species. Considering possible increasing risk of human *E. bieneusi* infection due to close contact with pet dogs and identification of zoonotic *E. bieneusi* genotypes, 589 fresh fecal specimens of pet dogs were collected from Yunnan Province, China to determine the occurrence of *E. bieneusi*, characterize dog-derived *E. bieneusi* isolates, and assess their zoonotic potential at the genotype level. *Enterocytozoon bieneusi* was identified and genotyped by PCR and sequencing of the internal transcribed spacer (ITS) region of the ribosomal RNA (rRNA) gene. Twenty-nine specimens (4.9%) were positive. A statistical difference was observed in occurrence rates of *E. bieneusi* in pet dogs among 11 sampling sites by Fisher’s exact test. Fifteen genotypes were identified and all of them phylogenetically belonged to zoonotic group 1, including four known genotypes (EbpC, D, Peru 8, and Henan-III) and 11 novel genotypes. Genotype Henan-III was reported in dogs for the first time. The finding of known genotypes found previously in humans and novel genotypes falling into zoonotic group 1 indicates that dogs may play a role in the transmission of *E. bieneusi* to humans in the investigated areas.

## Abstract (fr)

*Enterocytozoon bieneusi* est l’espèce de microsporidies la plus répandue chez l’homme et peut affecter plus de 200 espèces animales. Compte tenu du risque accru possible d’infection humaine à *E. bieneusi* en raison d’un contact étroit avec des chiens de compagnie et de l’identification de génotypes zoonotiques d’*E. bieneusi*, 589 échantillons fécaux frais de chiens de compagnie ont été collectés dans la province du Yunnan, en Chine, pour déterminer la présence d’*E. bieneusi*, caractériser les isolats obtenus de chiens, et évaluer leur potentiel zoonotique au niveau du génotype. *Enterocytozoon bieneusi* a été identifié et génotypé par PCR et séquençage de la région d’espacement transcrit interne (ITS) du gène de l’ARN ribosomal (ARNr). Vingt-neuf échantillons (4,9%) étaient positifs. Une différence statistique a été observée dans les taux de présence d’*E. bieneusi* chez les chiens de compagnie parmi 11 sites d’échantillonnage par le test exact de Fisher. Quinze génotypes ont été identifiés et tous appartenaient phylogénétiquement au groupe zoonotique 1, dont quatre génotypes connus (EbpC, D, Peru 8 et Henan-III) et 11 nouveaux génotypes. Le génotype Henan-III est signalé pour la première fois chez le chien. La découverte de génotypes connus précédemment trouvés chez l’homme et de nouveaux génotypes appartenant au groupe zoonotique 1 indique que les chiens peuvent jouer un rôle dans la transmission d’*E. bieneusi* aux humains dans les zones étudiées.

## Introduction

Microsporidia are obligate intracellular parasitic fungi, and they have the ability to infect both humans and animals. Within microsporidia, there are at least 1,700 recognized species belonging to approximately 220 genera, with 17 species having been found in humans [10]. Molecular epidemiological data have confirmed four major microsporidial species infecting humans, including *Enterocytozoon bieneusi*, *Encephalitozoon cuniculi*, *Encephalitozoon intestinalis*, and *Encephalitozoon hellem* [32]. Among them, *E. bieneusi* has been regarded as the most prevalent, accounting for more than 90% of human microsporidiosis cases [32]. According to the largest meta-analysis of microsporidia globally, the overall prevalence of *E. bieneusi* infection in humans was 7.9% [28]. Immunocompromized/immunodeficient individuals are reported to be the most susceptible population, such as HIV-positive patients, with the prevalence of *E. bieneusi* infection ranging from 1.2% to 100% [40]. *Enterocytozoon bieneusi* primarily invades the epithelial cells of the small intestine, and mainly causes disease characterized by diarrhea, and due to lack of effective drugs available, life-threatening diarrhea often occurs in immunocompromized patients [16]. *Enterocytozoon bieneusi* is also infrequently identified in other epithelial cells of patients with AIDS, *i.e.*, in the biliary tree, gallbladder, nonparenchymal liver cells, pancreatic duct, and tracheal, bronchial, and nasal epithelia [37]. In addition to humans, *E. bieneusi* has also been detected in at least 210 animal species, suggesting its zoonotic potential [15]. The findings of the same *E. bieneusi* genotypes in both humans and animals support the presumption of zoonotic potential. Meanwhile, in many epidemiological studies of *E. bieneusi* in animals, a high occurrence rate and a large percentage of zoonotic genotypes were observed, such as 93.7% and 100% in pigs in the Czech Republic [29], 37.6% and 100% in cattle in China [39], and 9.6% and 100% in dogs in Spain [9], implying a risk of zoonotic transmission. In fact, early in 2007 in Peru, Cama *et al.* reported zoonotic transmission of *E. bieneusi* based on the finding that an unusual genotype (Peru 16) was identified in seven guinea pigs and a 2-year-old child in the same household [2].

*Enterocytozoon bieneusi* transmission to humans is usually *via* the fecal-oral route through ingesting water and food contaminated by infective spores or direct contact with infected individuals (humans and animals) [27]. The epidemiological roles of water and food in the transmission of microsporidiosis caused by *E. bieneusi* are now well recognized. Three outbreaks related to *E. bieneusi* have been documented worldwide: one waterborne outbreak in France; and two foodborne outbreaks in Sweden and Denmark [4, 6, 23]. Due to clinical importance and public health significance, in the United States, the National Institute of Allergy and Infectious Diseases (NIAID) classified *E. bieneusi* as a Category B Priority Pathogen [8], and the Environmental Protection Agency (EPA) listed this microsporidian in its Contaminant Candidate List of Waterborne Organisms (https://www.epa.gov/ground-water-and-drinking-water/national-primary-drinking-water-regulations).

*Enterocytozoon bieneusi* is now known as a species complex. By analyzing sequences of the internal transcribed spacer (ITS) region of the ribosomal RNA (rRNA) gene with a high degree of genetic polymorphism, 819 genotypes had been identified by June 2021: 126 exclusively in humans, 614 exclusively in animals, 58 in both humans and animals, and 21 only in environmental samples [15]. These genotypes have been divided into 11 different phylogenetic groups and an outlier with a varying degree of host specificity and zoonotic potential [17]. The genotypes of groups 1 and 2 present a broad range of hosts including humans, suggesting low host specificity and large potential for zoonotic or cross-species transmission; in contrast, the genotypes of groups 3–11 and the outlier appear to be more host-specific, indicating limited zoonotic potential [17].

Dogs are common and useful animals. However, they can carry a variety of pathogens, including zoonotic *E. bieneusi*. Since the first report of *E. bieneusi* in dogs in Switzerland in 1999 [22], to date, more than 5,300 dogs from 9 countries have been involved in 21 epidemiological investigations of *E. bieneusi* worldwide, and the average prevalence was 10.9% (585/5363), with 1.7%–36.9% for stray dogs, 3.2%–17.9% for pet dogs, 0.8%–9.6% for household dogs, 3.3%–8.6% for clinic dogs, and 8.3% for farm dogs (Table 1) [1, 3, 5, 7, 9, 14, 18, 19–22, 25, 26, 30, 33, 36, 38, 39, 41–43].

**Table 1:** Prevalence and genotype distribution of *E. bieneusi* in dogs worldwide.

Source and Country	% (No. positive/No. examined)	Group	Genotype (*n*)	Zoonotic genotype (*n*)	% Zoonotic genotypes	References
Pet						
China	7.7 (2/26)	2	CHN5 (1), CHN6 (1)	CHN6 (1)	50.0	[39]
	11.7 (23/197)	1	D (2), macaque3 (2), CD2 (1), EbpC (1), Peru 8 (1), Type IV (1)	D (2), EbpC (1), Peru 8 (1), Type IV (1)	21.7	[14]
		2	CD6 (1)			
		11	PtEb IX (7), CD8 (4), CD7 (2), WW8 (1)			
	7.2 (18/249)	1	D (1), EbpC (1), EbpC+NED1 (1)^a^, NED2 (1)	D (1), EbpC (2)	14.3	[18]
		11	PtEb IX (12), PtEb IX+NED3 (1)^a^, PtEb IX+NED4 (1)^a^			
	5.9 (19/322)	1	D (1)	D (1)	5.3	[38]
		11	PtEb IX (18)			
	17.9 (90/502)	1	EbpC (7), D (6)	EbpC (7), D (6), I (2)	16.7	[33]
		2	I (2)			
		11	PtEb IX (31), GD2 (24), GD1 (10), WW8 (4), GD3 (2), GD4 (2), GD5 (1), GD6 (1)			
	6.3 (38/604)	1	EbpC (12), D (2), CD11 (1), CD12 (1), CD13 (1), Type IV (1)	EbpC (12), D (2), Type IV (1)	39.5	[3]
		11	PtEb IX (19), WW8 (1)			
	8.0 (21/262)	11	PtEb IX (18), DgEb I (1), DgEb II (1), WW8 (1)		0	[36]
	3.2 (2/62)	2	YCHA2 (1)		0	[43]
		11	YCHA3 (1)			
Japan	5.0 (1/20)	11	PtEb IX (1)		0	[1]
	4.4 (26/597)	11	PtEb IX (26)		0	[26]
Portugal	100 (3/3)^b^	1	D (1), Peru 6 (1)	D (1), Peru 6 (1)	66.6	[21]
		11	PtEb IX (1)			
Stray						
China	20.5 (31/151)	1	O (4), EbpA (2), CD1 (1), CD3 (1), CD4(1), D (1), PigEBITS5 (1)	O (4), EbpA (2), D (1), PigEBITS5 (1)	25.8	[14]
		7	CD5 (1)			
		11	PtEb IX (19)			
	8.8 (24/272)	1	Type IV (10), SHZJD1 (6), EbpA (3), SHZJD2 (1), SHZJD3 (1), SHZJD5 (1), HenanV+Type IV (1)^a^, HenanV+SHZJD4 (1)^a^	Type IV (10), EbpA (3), HenanV (1), HenanV+Type IV (1)	62.5	[20]
	18.8 (136/724)	1	SCD-2 (1), Type IV (1)	Type IV (1)	0.7	[42]
		11	WW8 (92), PtEb IX (41), SCD-1 (1)			
	39.6 (59/149)	1	D (6)	D (6)	10.2	[33]
		11	GD1 (28), PtEb IX (25)			
Colombia	15.0 (18/120)	1	Type IV (1), WL11 (1)	Type IV (1), WL11 (1)	11.1	[30]
Iran	5.3 (4/75)	1	D (4)	D (4)	100	[7]
Japan	1.7 (1/59)	11	PtEb IX (1)		0	[1]
Household						
Australia	4.4 (15/342)	1	D (5)	D (5)	33.3	[41]
		3	VIC_dog1 (1)			
		11	PtEb IX (9)			
China	7.8 (8/102)	11	PtEb IX (8)		3.4	[38]
Poland	4.9 (4/82)	1	D (2)	D (2)	50.0	[25]
		11	PtEb IX (2)			
Spain	9.6 (7/73)	1	A (7)	A (7)	100	[9]
	0.8 (2/237)	2	BEB6 (1)	BEB6 (1)	50.0	[5]
		11	PtEb IX (1)			
Clinic						
China	3.3 (2/61)	11	PtEb IX (2)		0	[38]
	8.6 (27/315)	1	EbpC (4), CHD1 (2), CHD2 (2)	EbpC (4)	14.8	[19]
		2	CM7 (3)			
		11	PtEb IX (16)			
Farm						
Switzerland	8.3 (3/36)	11	PtEb IX (3)		0	[22]

a Mixed infection of *E. bieneusi* genotypes was identified in these studies.

b Only *E. bieneusi*-positive isolates were genotyped.

In China, *E. bieneusi* as an emerging enteric pathogen was first reported in both humans and animals in 2011 [39]. To date, epidemiological studies of *E. bieneusi* in dogs have been carried out in 12 provinces and municipalities (Table 1). However, in southwestern China’s Yunnan Province, few data are available regarding *E. bieneusi* infection in dogs. Currently, there has been only one epidemiological study reporting occurrence and genotyping of *E. bieneusi* in dogs in Yunnan Province [36]. Considering that close human contact with pet dogs and identification of zoonotic genotypes in dogs can increase the risk of *E. bieneusi* infection, the present study was performed to determine the occurrence rate, genetic characterization, and zoonotic potential of *E. bieneusi* at the genotype level in pet dogs in Yunnan Province.

## Material and methods

### Ethics statement

The objective and procedure of the present study were reviewed and approved by the Research Ethics Committee and the Animal Ethics Committee of Hangzhou Medical College and the National Institute of Parasitic Diseases, Chinese Center for Disease Control and Prevention, China (IPD-2020-15). All work with animals was strictly performed in accordance with the Regulations for the Administration of Affairs Concerning Experimental Animals. Beginning work on the study, we contacted the pet dog owners and obtained their permission to have their animal feces involved in our study. All the animal fecal specimens were collected only after defecation without disturbing them.

### Fecal specimen collection

During a two-month period from April to June, 2021, 589 fresh fecal specimens (approximately 15 g) were collected from pet dogs (one specimen each) from 11 sampling sites distributing in eight cities/autonomous prefectures in Yunnan Province (Table 2). Each dog fecal specimen was collected immediately after defecation using disposable latex gloves and placed in clean sealed bags with their unique identification numbers on them. Meanwhile, ages of dogs and sampling sites were recorded. All the animals involved in the present study had no clinical signs of diarrhea at the time of sampling. The collected fecal specimens were shipped to our laboratory in a cooler with ice packs. If DNA extraction could be finished within 48 h, the fecal specimens were kept at 4 °C. If not, the fecal specimens were stored at −20 °C.

**Table 2:** Prevalence and genotypes of *E. bieneusi* in pet dogs in Yunnan Province.

City/autonomous prefecture	Sampling sites (N, E)^b^	Examined no.	Positive no. (%)	Genotype (n)	
				Known	Novel
Chuxiong Yi	Chuxiong (25.03°N, 101.55°E)	11	0		
Baoshan	Longyang (25.12°N, 99.17°E)	10	1 (10.0)	D (1)^b^	
Dali Bai	Heqing (26.55°N, 100.18°E)	5	1 (20.0)	EbpC (1)^b^	
	Jianchuan (26.54°N, 99.91°E)	54	3 (5.6)	D (2)^b^	YND-JC50 (1)
Diqing Tibetan	Deqin (28.47°N, 98.92°E)	51	0		
	Shangri-La (27.82°N, 99.71°E)	100	5(5.0)	EbpC (5)^b^	
Kunming	Shilin (24.81°N, 103.30°E)	54	0		
Lijiang	Gucheng (26.88°N, 100.23°E)	25	2 (8.0)	EbpC (1)^b^	YND-GC17 (1)
	Yulong (26.83°N, 100.24°E)	25	3 (12.0)	EbpC (1)^b^	YND-YL12 (1), YND-YL24 (1)
Nujiang Lisu	Lushui (25.98°N, 98.82°E)	204	6 (2.9)	EbpC (2)^b^, D (2)^b^, Peru8 (1)^b^	YND-LS10 (1)
Zhaotong	Zhaoyang (27.33°N, 103.72°E)	50	8 (16.0)	EbpC (1)^b^, Henan-III (1)^b^	YND-ZY12 (1), YND-ZY13 (1), YND-ZY15 (1), YND-ZY21 (1), YND-ZY36 (1), YND-ZY42 (1)
Total		589	29 (4.9)	EbpC (11)^b^, D (5)^b^, Peru8 (1)^b^, Henan-III (1)^b^	YND-GC17 (1), YND-JC50 (1), YND-LS10 (1), YND-YL12 (1), YND-YL24 (1), YND-ZY12 (1), YND-ZY13 (1), YND-ZY15 (1), YND-ZY21 (1), YND-ZY36 (1), YND-ZY42 (1)

a N and E indicate north latitude and east longitude, respectively.

b *E. bieneusi* genotypes identified in humans.

### DNA extraction

Genomic DNA was extracted from 180 to 200 mg of fecal specimens using a QIAamp DNA Stool Mini Kit (QIAGEN, Hilden, Germany), following the manufacturer’s instructions. To obtain a high yield of DNA, the lysis temperature was increased to 95 °C. DNA was eluted in 200 μL of ATE and stored at −20 °C before it was used for PCR analysis.

### PCR amplification

All the DNA preparations were analyzed for the presence of *E. bieneusi* by nested PCR amplification of a 410 bp fragment including the ITS region (243 bp) of the rRNA gene, as previously described [24]. Each specimen was subjected to at least two PCR reactions. A positive control (DNA of a human-derived genotype Type IV) and a negative control (nuclease-free water) were included in each PCR test. The secondary PCR products were analyzed by 1.5% agarose gel and visualized under UV by staining the gel with GelStrain (TransGen Biotech., Beijing, China).

### Nucleotide sequencing and analyzing

All expected size secondary PCR products were directly sequenced on an ABI PRISM 3730XL DNA Analyzer by Comate Bioscience Company Limited (Jilin, China), using the BigDye Terminator v3.1 Cycle Sequencing Kit (Applied Biosystems, Carlsbad, CA, USA). Accuracy of nucleotide sequences could be guaranteed by sequencing in both directions and by sequencing another two new PCR products for some of the DNA preparations yielding new nucleotide sequences.

The raw sequences obtained in the present study were spliced together with Clustal X 1.81 (http://www.clustal.org/) and aligned with sequences deposited in the GenBank database by BLAST analysis to determine *E. bieneusi* genotypes. All the genotypes were identified only based on 243 bp of the ITS region of the rRNA gene of *E. bieneusi*, according to the established nomenclature system [31]. If the sequences obtained in the present study were identical to the previous published sequences, they were determined as known genotypes and given the first published genotype names. In contrast, if the sequences had single nucleotide substitutions, deletions or insertions compared to the sequences of the known genotypes, they were considered novel genotypes and would be given their names.

### Phylogenetic analysis

To analyze the genetic relationship between *E. bieneusi* genotypes identified in the present study and those described in previous studies, a neighbor-joining (NJ) tree of the ITS sequences was constructed using Molecular & Evolution Genetic Analysis software version 11.0 (MEGA 11.0) (http://www.megasoftware.net/) based on the evolutionary distances calculated by the Kimura-2-parameter model. Bootstrap analysis with 1,000 replicates was used to determine support for the clades.

### Statistical analysis

Fisher’s exact test implemented in Statistical Package for the Social Sciences (SPSS) version 23.0 software was used to compare differences in occurrence rates of *E. bieneusi* in animals from different sampling sites. A *p* value of < 0.05 was considered statistically significant.

## Results

### Occurrence rate of *E. bieneusi* in pet dogs

A total of 589 fecal specimens from pet dogs were tested for the presence of *E. bieneusi* by PCR amplification of the ITS region of the rRNA gene. In all, 29 specimens (4.9%, 29/589) were PCR-positive and all of them were identified to be *E. bieneusi* by sequence analysis. *Enterocytozoon bieneusi* was found in pet dogs from eight areas: Heqing (20.0%, 1/5), Zhaoyang (16.0%, 8/50), Yulong (12.0%, 3/25), Longyang (10.0%, 1/10), Gucheng (8.0%, 2/25), Jianchuan (5.6%, 3/54), Shangri-La (5.0%, 5/100) and Lushui (2.9%, 6/204). There was an absence of *E. bieneusi* in Chuxiong, Deqin, and Shilin. A statistical difference was observed in occurrence rates of *E. bieneusi* in pet dogs from different sampling sites (*p* = 0.002) (Table 2).

### Genetic characterization of the ITS region of the rRNA gene

Based on sequence analysis of the ITS region of the rRNA gene of 29 *E. bieneusi* positive isolates, 15 genotypes were identified with 26 polymorphic sites being observed among them (Table 3). Four genotypes were known, including genotypes EbpC (*n* = 11), D (*n* = 5), Peru 8 (*n* = 1), and Henan-III (*n* = 1). The remaining 11 genotypes were novel, named YND-GC17, YND-JC50, YND-LS10, YND-YL12, YND-YL24, YND-ZY12, YND-ZY13, YND-ZY15, YND-ZY21, YND-ZY36, and YND-ZY42 (one each) (GenBank: OR750543–OR750553) (Table 2). They had the largest similarity with genotypes EbpC, Henan-IV, PigSpEb2, NCM-1, NCM-2, and CS-1 of group 1. Detailed results of homology analysis of the novel *E. bieneusi* genotypes in the ITS region of the rRNA gene are summarized in Table 4.

**Table 3:** Variation at 26 polymorphic sites within the ITS region of the rRNA gene of *E. bieneusi* isolates obtained in this study.

Genotype	Accession No.	Nucleotide at position																									
		12	14	23	28	31	41	50	51	52	74	81	93	95	109	113	117	132	141	146	154	158	173	189	196	201	224
EbpC	AF076042	G	T	C	T	G	A	T	G	T	A	C	T	G	T	T	G	T	C	G	C	T	G	A	A	T	G
D	LC436479	–	–	–	–	–	–	–	–	–	–	–	C	–	–	C	T	–	T	–	–	–	–	–	–	–	–
Peru8	MN747470	–	–	–	–	–	–	–	–	–	–	–	C	–	–	C	–	–	T	–	–	–	–	–	–	–	–
Henan-III	MN179308	–	–	–	–	–	–	–	/	/	–	–	–	–	–	–	–	–	–	–	–	–	–	–	–	–	–
YND-GC17	OR750543	–	–	T	–	–	–	–	–	–	–	–	–	–	–	–	–	–	–	–	–	–	–	–	–	–	–
YND-JC50	OR750544	–	–	–	–	–	–	–	–	–	–	–	–	–	–	–	–	–	–	–	/	–	–	–	–	–	–
YND-LS10	OR750545	A	–	–	–	–	–	–	–	–	–	–	–	–	–	–	–	–	–	A	–	–	–	–	–	–	–
YND-YL12	OR750546	–	–	–	–	–	–	C	–	–	G	–	–	–	–	–	–	–	–	–	–	–	A	–	G	–	–
YND-YL24	OR750547	–	C	–	–	–	–	–	–	–	–	–	–	–	–	–	–	–	–	–	–	–	–	–	–	–	–
YND-ZY12	OR750548	–	–	–	–	–	–	–	–	–	–	–	–	–	–	–	–	C	–	–	–	–	A	–	G	–	–
YND-ZY13	OR750549	–	–	–	C	A	–	–	–	–	–	T	–	T	G	–	–	–	–	–	–	–	A	–	G	–	–
YND-ZY15	OR750550	–	–	–	–	–	–	–	–	–	–	–	–	–	–	–	–	–	–	–	–	–	–	–	–	C	–
YND-ZY21	OR750551	–	–	–	–	–	–	–	–	–	–	–	–	–	–	–	–	–	–	–	–	–	A	G	G	–	–
YND-ZY36	OR750552	–	–	–	–	–	G	–	–	–	–	–	–	–	–	–	–	–	–	–	–	C	–	–	–	–	A
YND-ZY42	OR750553	–	–	–	–	–	–	–	–	–	–	–	–	–	–	–	–	–	–	–	–	–	A	–	G	–	–

The symbol “/” denotes base deletion.

The symbol “–” denotes sequence identity to genotype EbpC (AF076042).

**Table 4:** Homology analysis of the novel *E. bieneusi* genotypes in the ITS region of the rRNA gene.

Genotype^a^ (*n*)	Accession No.^a^	Genotype (accession No.^b^)	Homology (%)	Nucleotide (position)
YND-GC17 (1)	OR750543	EbpC (MN758739)	99.6	C to T (23)
YND-JC50 (1)	OR750544	EbpC (MN758739)	99.6	C to – (154)
YND-YL24 (1)	OR750547	EbpC (MN758739)	99.6	T to C (14)
YND-ZY15 (1)	OR750550	EbpC (MN758739)	99.6	T to C (201)
YND-LS10 (1)	OR750545	Henan-IV (MK681472)	99.6	G to A (146)
YND-YL12 (1)	OR750546	PigSpEb2 (OM219033)	98.8	T to C (50); A to G (74); G to A (173)
YND-ZY12 (1)	OR750548	PigSpEb2 (OM219033)	99.2	T to C (132); G to A (173)
YND-ZY42 (1)	OR750553	PigSpEb2 (OM219033)	99.6	G to A (173)
YND-ZY21 (1)	OR750551	PigSpEb2 (OM219033)	99.2	G to A (173); A to G (189)
		NCM-2 (MF440666)	99.2	G to A (173); A to G (196)
YND-ZY13 (1)	OR750549	CS-1 (KU194593)	98.8	T to C (28); T to G (109); G to A (173)
YND-ZY36 (1)	OR750552	NCM-1 (MF440665)	99.2	T to C (158); G to A (224)

The dash “–” denotes base deletion.

a Accession Nos. representing sequences obtained in the present study for the first time.

b Accession Nos. representing the published sequences with the largest similarity to the sequences obtained in the present study.

### Geographical distribution of *E. bieneusi* genotypes

*Enterocytozoon bieneusi* was found in dogs from eight sampling sites. Four known genotypes (EbpC, D, Peru 8, and Henan-III) were found at six, three, one and one sites, respectively with genotypes EbpC, D, and Peru 8 appearing at one site (Shangri-La). The 11 novel genotypes were from five areas, with 5 appearing in Zhaoyang (Table 2). Genotype EbpC was observed to have the widest geographical distribution.

### Phylogenetic relationship of *E. bieneusi* genotypes

**Figure 1:**
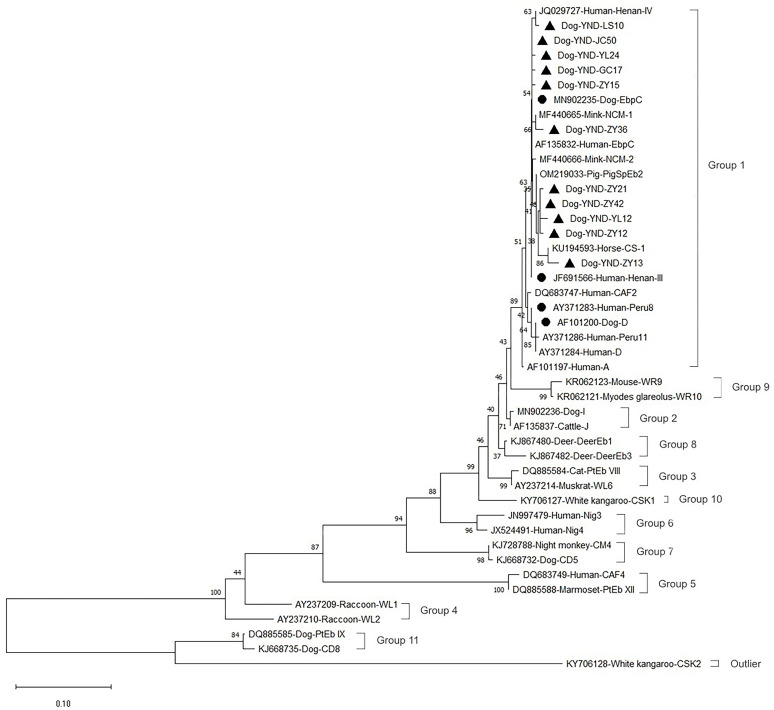
Phylogenetic relationships between genotypes of *Enterocytozoon bieneusi* identified in this study and known genotypes deposited in GenBank, as inferred by a neighbor-joining analysis of the ITS rRNA gene sequences based on genetic distances calculated by the Kimura-2-parameter model. Numbers on the branches are percent bootstrapping values from 1,000 replicates. Each sequence in this figure is identified by its accession number, host origin, and genotype designation. The group terminology for the clusters is based on the work of Li *et al.* (2019). Before the genotype names, the circles and triangles filled in black indicate the known and novel genotypes identified in present study, respectively.

In a phylogenetic analysis of a neighbor-joining tree of the ITS sequences of the rRNA gene of *E. bieneusi*, 15 genotypes (four known genotypes and 11 novel genotype) obtained in the present study all fell into group 1, considered to be the zoonotic group (Figure 1).

## Discussion

It is already known that dogs can provide many services to humans. Recently, it has been evidenced that a dog companion may be good for our physical health, since contact with dogs can lower blood pressure, ameliorate depression, and even produce a survival benefit after myocardial infarction [12]. Pet popularity has been increasing over the past few years. According to the Chinese Pet Industry White Paper, the number of pet dogs has increased rapidly to have reached 62 million up to 2019 (https://www.chyxx.com/industry/202006/874331.html). However, dogs can act as reservoir hosts for many zoonotic pathogens, and they can transmit the diseases to humans. Thus, dog health is not only a veterinary issue, but also a public health issue. In the investigated area (Yunnan Province), dogs were reported to carry at least 40 pathogens, with 24 of them being zoonotic [11]. A recent epidemiological investigation reported *E. bieneusi* in dogs in Yunnan Province in 2021 for the first time [36].

In the present study, 4.9% (29/589) of pet dogs were infected with *E. bieneusi*. The occurrence rate was lower than the global average occurrence (8.4%) of *E. bieneusi* in pet dogs and was only slightly higher than those reported in pet dogs in two studies worldwide: 4.4% in Japan and 3.2% in Heilongjiang Province of northeast China (Table 1). The low occurrence rate of *E. bieneusi* in pet dogs in the present study might be attributable to the fact that all the fecal specimens were collected from adult dogs without clinical signs of illness. In fact, occurrence rates of *E. bieneusi* in dogs are affected by many factors. Epidemiological data indicated that stray dogs had the highest rate (17.6%, 273/1,550) of *E. bieneusi* compared to pet dogs (8.5%, 243/2,844), farm dogs (8.3%, 3/36), dogs from clinics or pet hospitals (7.7%, 29/376), and household dogs (6.5%, 36/556) (Table 1). This might be because the investigated stray dogs were usually housed at dog shelters and raised in a crowded house or enclosure, inevitably leading to occurrence of cross-transmission/infection of *E. bieneusi* between different individuals. *Enterocytozoon bieneusi* is a causative agent of opportunistic infection. The health status of hosts seems to be the key factor related to *E. bieneusi* infection, and it has been confirmed to be closely associated with host age. In a study of molecular detection of *E. bieneusi* in pet dogs in Japan, a significant difference in occurrence rates was found between two age groups (<1 year: 8.3% *versus* ≥1 year: 3.4%) [26]. In another study of *E. bieneusi* in dogs in Australia, juvenile dogs were significantly associated with a higher occurrence rate of *E. bieneusi* than adult dogs [41]. It is understandable that hosting inversely associates with occurrence rates of *E. bieneusi*, because the young animals usually have immature immune systems. In general, occurrence rates are complicated and difficult to compare.

Due to a high degree of genetic polymorphism of the ITS region of the rRNA gene of *E. bieneusi*, sequence analysis of this region is currently considered the standard method for genotyping *E. bieneusi* isolates [31]. Previous epidemiological studies of *E. bieneusi* in dogs have identified 54 genotypes, and most of them belonged to zoonotic group 1 (*n* = 29), group 2 (*n* = 7), and dog specific group 11 (*n* = 16). There were another two genotypes identified, with one (CD5) in group 3, and the other (VIC_dog1) in group 7 (Table 1). Among them, 14 of 54 genotypes have been identified in humans. Therefore, dogs infected with *E. bieneusi* might pose a threat to human health. In the present study, sequence analysis of the ITS region identified 15 genotypes in 29 *E. bieneusi* isolates, including 4 known genotypes (D, EbpC, Peru 8, and Henan-III) and 11 novel genotypes.

Genotypes D (syn. CEbC, NCF7, Peru 9, PigEBITS9, PtEb VI, SHW1, WL8) and EbpC (syn. CHG23, E, Peru 4, SC03, WL13, WL17) were the most common genotypes either in humans or in animals worldwide [15]. To date, genotype D has been identified in 91 host species distributed in 40 countries, while genotype EbpC has been identified in 43 host species in 15 countries. They both showed a wide host range and geographical distribution of the two genotypes [40]. In China, genotypes D and EbpC have been detected in humans and numerous wild, domestic, and companion animals from 26 provinces/municipalities [35]. Meanwhile, human *E. bieneusi* infection cases caused by genotypes D and EbpC were ranked first and second, respectively [13]. In addition, we also identified zoonotic genotypes Peru 8 (syn. CQR-1) and Henan-III (syn. BLC17), with genotype Henan-III being reported in dogs for the first time worldwide. In comparison with genotypes D and EbpC, the two genotypes seemed to have a relatively narrow host range and geographical distribution. Based on current epidemiological data of *E. bieneusi* summarized in a review article by Koehler et al. and the present genotyping data, to date, genotype Peru 8 has only been identified in 15 host species in 7 countries (China: 11 animal species), while genotype Henan-III has only been identified in 6 host species in 2 countries (China: 5 animal species) [7]. In China, human microsporidiosis cases caused by genotypes Peru 8 (*n* = 1) and Henan-III (*n* = 1) were only found in HIV-positive patients on antiretroviral therapy in Henan Province [34]. The finding of four zoonotic genotypes (D, EbpC, Peru 8, and Henan-III) in dogs indicates that the epidemiological role of dogs in the transmission of human microsporidiosis caused by *E. bieneusi* needs to be given more attention. Meanwhile, pet owners should also be made aware of the potential zoonotic transmission of microsporidiosis due to close contact with infected animals.

With accumulation of genotyping data of *E. bieneusi*, there has been a huge number of genotypes identified. By June 2021, 819 genotypes had been documented, with 465 in China [15], revealing the vast genetic variation of *E. bieneusi* in the ITS region of the rRNA gene. In the present study, 11 novel genotypes of *E. bieneusi* were obtained, and all of them fell into zoonotic group 1. Meanwhile, in a homology analysis, five novel genotypes had the largest similarity (99.6%) with two zoonotic genotypes (EbpC and Henan-IV), suggesting their zoonotic potential.

## Conclusion

In conclusion, the present study demonstrated occurrence (4.9%, 29/589) and genetic characterization of *E. bieneusi* in the ITS region of the rRNA gene in pet dogs in Yunnan Province, China. The finding of four zoonotic genotypes in dogs indicates possible occurrence of dog-related zoonotic transmission of human microsporidiosis caused by *E. bieneusi* in the investigated area. Meanwhile, the observation of all the 11 novel genotypes falling into zoonotic group 1 implies broad zoonotic potential and public health significance. Since the investigated dogs in the present study were asymptomatic, the health condition of these animals was usually neglected. Once pet dogs are infected with *E. bieneusi*, they have more time and opportunity to continually release infective *E. bieneusi* spores with feces into the environment to spread the parasitic disease to humans. Furthermore, the high frequency of human contact with pet dogs increases the risk of *E. bieneusi* infection. Currently, due to no safe and effective drug treatment and vaccines prevention against microsporidiosis caused by *E. bieneusi*, it is particularly important to develop control strategies to intervene with and prevent human infection. Thus, advice should be given to pet owners, including adequate hygiene practices (adequate pet waste disposal and regular hand washing) and routine veterinary care. Health education should also be provided to pet owners to increase their risk awareness of the potential zoonotic transmission of *E. bieneusi*.
